# Disruption of Foveal Space Impairs Discrimination of Peripheral Objects

**DOI:** 10.3389/fpsyg.2016.00699

**Published:** 2016-05-11

**Authors:** Kimberly B. Weldon, Anina N. Rich, Alexandra Woolgar, Mark A. Williams

**Affiliations:** ^1^Perception in Action Research Centre, Department of Cognitive Science, Faculty of Human Sciences, Macquarie UniversitySydney, NSW, Australia; ^2^ARC Centre of Excellence in Cognition and its Disorders, Macquarie UniversitySydney, NSW, Australia

**Keywords:** visual perception, periphery, feedback, fovea, distractor

## Abstract

Visual space is retinotopically mapped such that peripheral objects are processed in a cortical region outside the region that represents central vision. Despite this well-known fact, neuroimaging studies have found information about peripheral objects in the foveal confluence, the cortical region representing the fovea. Further, this information is behaviorally relevant: disrupting the foveal confluence using transcranial magnetic stimulation impairs discrimination of peripheral objects at time-points consistent with a disruption of feedback. If the foveal confluence receives feedback of information about peripheral objects to boost vision, there should be behavioral consequences of this phenomenon. Here, we tested the effect of foveal distractors at different stimulus onset asynchronies (SOAs) on discrimination of peripheral targets. Participants performed a discrimination task on target objects presented in the periphery while fixating centrally. A visual distractor presented at the fovea ~100 ms after presentation of the targets disrupted performance more than a central distractor presented at other SOAs. This was specific to a central distractor; a peripheral distractor at the same time point did not have the same effect. These results are consistent with the claim that foveal retinotopic cortex is recruited for extra-foveal perception. This study describes a new paradigm for investigating the nature of the foveal feedback phenomenon and demonstrates the importance of this feedback in peripheral vision.

## Introduction

The traditional model of visual object recognition is a primarily feedforward process in which visual input is processed in successive stages that correspond to the functional architecture of the ventral visual stream (Riesenhuber and Poggio, 1999). Feedback connections from higher-order cortical areas to lower-order areas, however, are a significant feature of the primate visual cortex (Felleman and Van Essen, 1991) and there is growing evidence that feedback plays a critical role in visual perception (for a review, see Panichello et al., 2012). Recent evidence from studies using transcranial magnetic stimulation (TMS) point to the crucial role of feedback signals from high to low visual areas, for example in perceptual completion (Wokke et al., 2012), conscious perception of motion (Silvanto et al., 2005), and scene categorization (Koivisto et al., 2011).

Established theories propose that feedback acts by modulating or anticipating pre-activated feedforward visual input (Supèr et al., 2001; DiCarlo et al., 2012). Evidence from functional magnetic resonance imaging (fMRI) suggests category information about objects in the periphery is fed back to foveal confluence (Williams et al., 2008). In that study, participants fixated centrally while performing a discrimination task on novel objects from different categories (“spiky,” “smoothie,” and “cubie” novel objects; Op de Beeck et al., 2006) in the periphery. Using multivoxel pattern analysis (MVPA), the authors were able to decode information about object category from foveal retinotopic cortex, an area distinct from the feedforward location of visual input. Importantly, this representation seemed to be behaviorally relevant: stronger representation in foveal retinotopic cortex correlated with better performance on the task. While it is difficult to determine precisely which category features are being encoded at the foveal confluence, the results of Williams et al. (2008) demonstrate a feedback mechanism that does something different than modulate existing activity: it constructs a new and separate representation.

In a subsequent study, Chambers et al. (2013) used TMS to disrupt the foveal confluence during discrimination of peripheral novel objects to test whether foveal cortex plays a critical role in perception of objects in the periphery or if it is simply an artifact of peripheral perception. TMS stimulation applied to foveal confluence approximately 350–400 ms *after* stimulus onset impaired discrimination of peripheral objects, a time too late to attribute to interference with the feedforward timecourse of the visual system. TMS applied at stimulus onset asynchronies (SOAs) from 150 ms prior to stimulus onset to 250 ms post-stimulus onset, as well as beyond 400 ms post-stimulus onset, had no such effect. The authors concluded that feedback to foveal retinotopic cortex is critical for perception of peripheral objects.

The aim of the present study was to design a behavioral paradigm to test the consequences of feedback about peripheral objects to foveal cortex by causing this feedback to compete with feedforward information from a visual distractor stimulus displayed at fixation. Whereas a TMS pulse has a fairly immediate effect on its targeted cortical sites, a visual distractor must go through the usual feedforward processes of the visual system (~40–120 ms; Kammer, 2007). We therefore selected a series of SOAs for the onset of the distractor relative to the peripheral targets including an SOA equivalent to the point at which TMS was effective, taking into account the feedforward processing time, to explore the timecourse of any effects. We also conducted a location-control experiment to test for the specificity of any effects to the fovea. We hypothesized that a central visual distractor appearing after target onset would disrupt behavioral performance more than visual distractors appearing at other SOAs, and that this would be specific to a foveal distractor.

With this paradigm, we were also able to explore whether the similarity of the distractor to peripheral targets affected performance. There is evidence that the characteristics of a visual distractor presented at the fovea can modulate its effect on visual search performance. In a study by Beck and Lavie (2005), participants were instructed to identify which of two pre-specified target letters (*X* or *N*) appeared in a circular array of non-target letters while either a compatible (identical to the target) or neutral (*S*, never included in the array of non-targets) distractor appeared simultaneously at fixation. Participants were slower to respond and made more errors when the distractor at fixation was neutral than when it was compatible, demonstrating that the degree of similarity between targets and distractors affected performance. Therefore, we also tested whether the similarity of the distractor present at fixation to peripheral targets likewise affected behavioral performance by presenting two types of distractors, either consistent or inconsistent with the category of the peripheral targets.

To preempt our results, we show a selective impairment of peripheral object discrimination by a central distractor presented at 117 ms post-target onset relative to other SOAs. The type of the distractor (consistent or inconsistent in object category with the targets) did not influence the pattern of results. The effect was both time- and location-specific: a distractor presented in the periphery had a similar effect at every SOA; in contrast, a centrally presented distractor had a greater disruptive effect at the critical SOA. These data are consistent with the idea that the foveal representation in visual cortex is recruited to assist with challenging tasks on peripheral stimuli.

## Experiment 1

### Materials and Methods

#### Participants

Twenty participants were recruited for Experiment 1. Nineteen participants (12 female; mean age = 24.58 years, *SD* = 8.31) completed the experiment and one participant’s data were discarded due to chance-level performance, leaving 18 full datasets for analysis. Participants received either course credit or $15 for their participation. All participants reported normal or corrected-to-normal visual acuity and gave informed consent. This study was approved by the Macquarie University Human Research Ethics Committee (Medical Sciences).

#### Stimuli and Apparatus

Sixteen stimuli were selected from a set of 1296 pre-generated “smoothie” and “cubie” stimuli (Op de Beeck et al., 2006). The main stimuli were 16 smoothie exemplars, selected to represent the more extreme variations from the larger set. A further smoothie stimulus, which was not one of the 16 main exemplars, was selected for use as a “category consistent” visual distractor in both experiments. A single “cubie” stimulus was selected for use as a “category inconsistent” distractor in Experiment 1. Viewing distance was maintained at 54 cm by use of a padded chinrest. At this distance, all stimuli subtended ~1.5° visual angle in width and the peripheral stimuli were presented 6.5° from fixation.

Experimental sessions took place in a dimly lit, windowless laboratory at Macquarie University, Sydney. Stimuli were presented on a 27″ Samsung SyncMaster AS950 monitor at a resolution of 1920 pixels × 1080 pixels and a refresh rate of 120 Hz.

#### Procedure

##### Training

**FIGURE 1:**
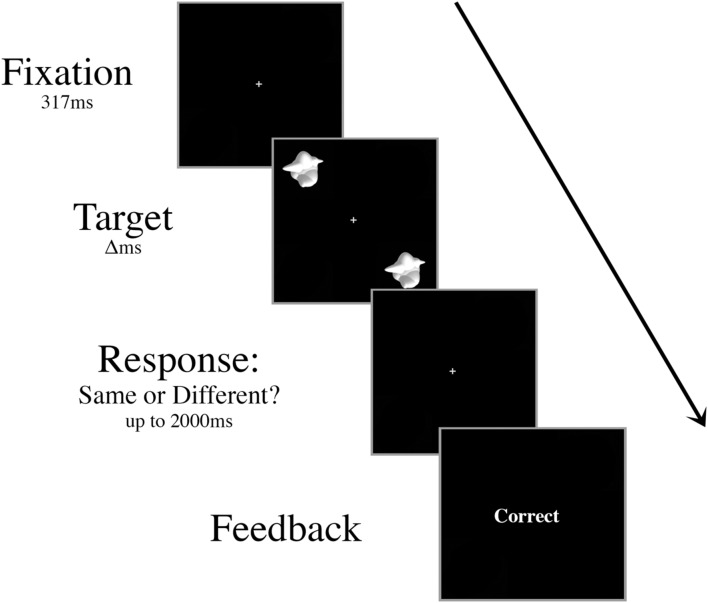
**Schematic of an example trial in the basic discrimination training task.** Targets were presented for decreasing durations (Δ, 417 ms, 267 ms, then 117 ms) during training until the participant was able to perform at 70% accuracy with a 117 ms presentation time.

Prior to the experiment, participants were trained on a basic discrimination task. Two smoothies were displayed for 417 ms in the upper left and lower right quadrants of the screen (**Figure 1**). In half of the trials, these target stimuli were different smoothies randomly selected from the set of 16 exemplars, and in the other half they were identical smoothies. Participants fixated centrally and responded with their right index finger to indicate a “same” judgment or their right middle finger to indicate a “different” judgment. Following each response, participants were given onscreen accuracy feedback. The interstimulus interval was 2000 ms.

Participants were instructed to maintain fixation on the central cross throughout each trial and respond as quickly and accurately as possible. We tracked fixation using eye-tracking of the right eye at 500 Hz with an Eyelink 1000 remote eye-tracker. The camera and infrared illuminator was mounted in front of the participant below the desktop display so that the screen was not obscured. Trials where the participant’s eye gaze drifted more than 2 from the center of the display were discarded.

Trials were presented in blocks of ten. Once participants could perform the discrimination task with >70% accuracy in a single block, the presentation time of the targets decreased to 267 ms. The presentation time of the targets further decreased to 117 ms once participants were able to perform the task with >70% accuracy in a block. Training continued until participants were able to make at least 70% correct discriminations when the target array was displayed for 117 ms, while maintaining fixation throughout the block.

##### Experiment 1

The design for Experiment 1 was very similar to the basic discrimination task, with the addition of the distractor stimulus and a standard duration for the target presentation. At the beginning of each trial, a white central cross was displayed for 567 ms. Participants were asked to fixate on the white cross throughout each trial. In each target display, two smoothies were displayed for 117 ms in opposite diagonal locations, each at 6.5° eccentricity. As in the training task, in half the trials the targets were different smoothies, randomly selected from the set of 16 exemplars, and in the other half they were identical. At one point in the trial, a distractor object appeared at fixation for 117 ms. Although we did not use eye-tracking in the main experiment, participants were trained to maintain central fixation in the training task, and the short duration of the targets in disparate locations made eye-movements counterproductive.

Participants were given 3 s to respond after the completion of the trial before the next trial automatically commenced. As in the basic discrimination task, participants used their right index finger to indicate a “same” judgment or their right middle finger to indicate a “different” judgment. Following each response, participants were given onscreen accuracy feedback.

**FIGURE 2:**
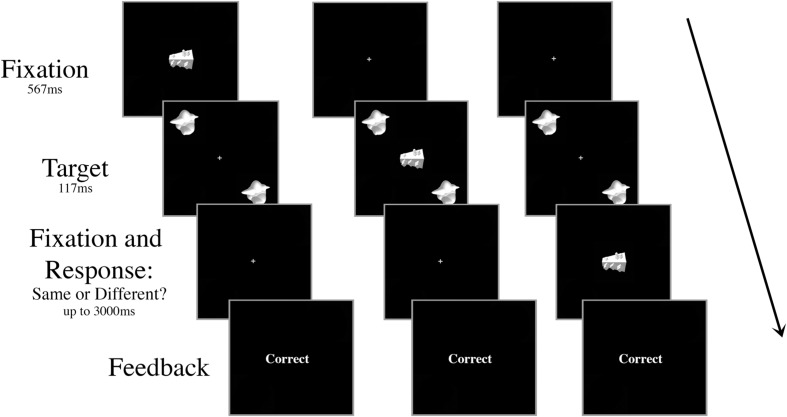
**Schematic of three example trials in Experiment 1 with an inconsistent distractor.** The targets and the distractor were displayed for 117 ms regardless of SOA. The distractor appeared either **(A)** 267 ms or 117 ms prior to target onset, **(B)** simultaneously with target onset, or **(C)** 117 ms or 267 ms after target onset. In the example shown, the targets are identical and the correct response is “same.”

There were ten trial conditions that dictated the timing and the type of the distractor presented, all randomly intermingled and fully crossed. First, the onset of the distractor object occurred at one of five possible SOAs: 267 ms prior to target onset (-267 ms), 117 ms prior to target onset (-117 ms), simultaneously with target onset (0 ms), 117 ms after target onset (+117 ms), or 267 ms after target onset (+267 ms). Second, the distractor was either consistent (smoothie) or inconsistent (cubie) with the category of the targets (50% consistent, 50% inconsistent; **Figure 2**). There were 80 trials for each of the ten conditions (40 “same,” 40 “different”) for a total of 800 trials in a session. All of the trial types were intermingled randomly, but blocked to give the participant a break after every 100 trials.

We did not include a non-distractor condition because the participants were trained without a distractor in the basic discrimination task and therefore they could have an advantage in a non-distractor condition simply due to practice. Additionally, the presence of a distractor introduces variables in the task that would not be well matched to a non-distractor condition. Instead, we used performance in the -267 ms SOA condition as a baseline for comparison.

### Results

Our dependent variable was *d′* for target discrimination. The hit rate was defined as the proportion of correct “same” responses on “same” trials, and the false alarm rate was defined as the proportion of “same” responses on “different” trials. A two-way repeated measures ANOVA on *d′* with the factors of distractor type (consistent, inconsistent) and SOA (-267 ms, -117 ms, 0 ms, +117 ms, and +267 ms) revealed a significant main effect of SOA [*F*(4,68) = 10.38, *p* < 0.001] but no main effect of distractor type [*F*(1,17) = 2.842, *p* = 0.11] and no interaction [*F*(4,68) < 1].

**FIGURE 3:**
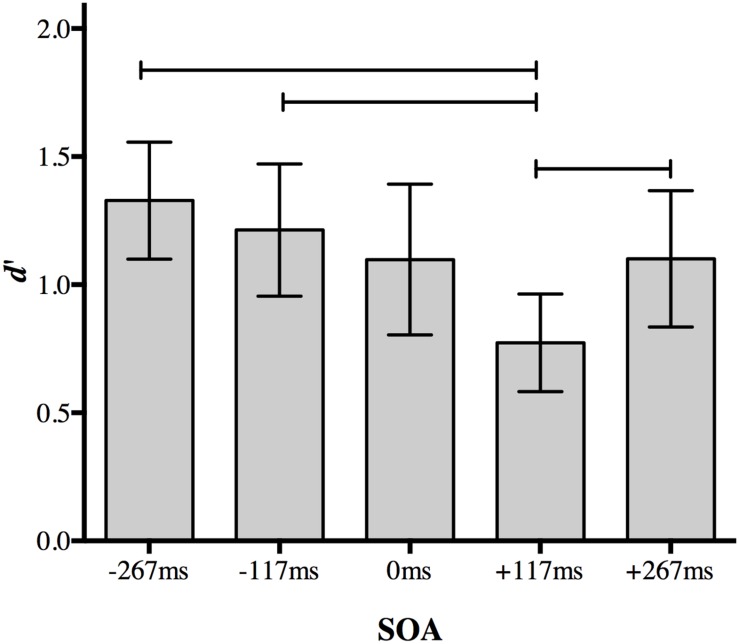
**The effect of a foveal distractor on peripheral target discrimination in Experiment 1.** A distractor appearing 117 ms after target onset disrupted discrimination more than distractors appearing at non-simultaneous SOAs. Lines indicate statistically significant differences between means after Bonferroni correction for 10 comparisons (*p* < 0.005). Error bars represent 95% confidence intervals.

A Bonferroni correction for multiple comparisons (α = 0.05/10 = 0.005) was applied to *post hoc* analyses following up the main effect of SOA (data collapsed over distractor type). A central distractor appearing at +117 ms SOA disrupted participants’ ability to discriminate between the peripheral targets relative to other non-simultaneous SOAs. Perceptual accuracy was impaired when the distractor was presented at +117 ms SOA compared to a distractor presented at 267 ms SOA (*p* < 0.001), -117 ms SOA (*p* = 0.001), and +267 ms SOA (*p* = 0.002; **Figure 3**). Accuracy for +117 ms SOA was marginally impaired relative to simultaneous presentation (*p* = 0.005).

No other comparison approached significance after correction. Discrimination accuracy did not differ significantly when a distractor appeared at the earliest SOA (-267 ms) compared to a distractor appearing at -117 ms (*p* = 0.077), 0 ms (*p* = 0.022), or +267 ms (*p* = 0.015). Discrimination accuracy also did not differ significantly between at distractor at 117 ms SOA and 0 ms SOA (*p* = 0.17) or +267 ms SOA (*p* = 0.22), nor between 0 and +267 ms SOA (*p* = 0.977).

The difference between performance at +117 and +267 ms SOA demonstrates the temporal specificity of the effect: it is not simply due to a distractor following the targets. Thus, the presence of an irrelevant object presented at fixation during the 117–234 ms post-target time period selectively disrupts discrimination of peripheral objects even relative to a distractor presented simultaneously with the targets. There was no evidence for an effect of distractor object category.

## Experiment 2

The aim of Experiment 2 was to see whether the effect of the foveal distractor in Experiment 1 is specific to the foveal location, as predicted by the foveal feedback interpretation, or if the result reflects more general effects of having a distractor presented. For example, there may be an overall alerting effect of having a stimulus presented just prior to the targets (-267 ms or -117 ms SOA), general impairment due to increased clutter in the display (0 ms SOA, although potentially this is greater for foveal than peripheral distractors), or some impact on memory of the targets or response selection (+117 ms and +267 ms SOA).

### Materials and Methods

#### Participants

A naïve group of 20 participants was recruited for Experiment 2 (12 female; mean age = 20.16 years, *SD* = 2.87). One participant’s dataset was discarded for chance performance, leaving 19 full datasets for analysis. Participants received either course credit or $15 for their participation. All participants reported normal or corrected-to-normal visual acuity and gave informed consent. This study was approved by the Macquarie University Human Research Ethics Committee (Medical Sciences).

#### Stimuli and Apparatus

**FIGURE 4:**
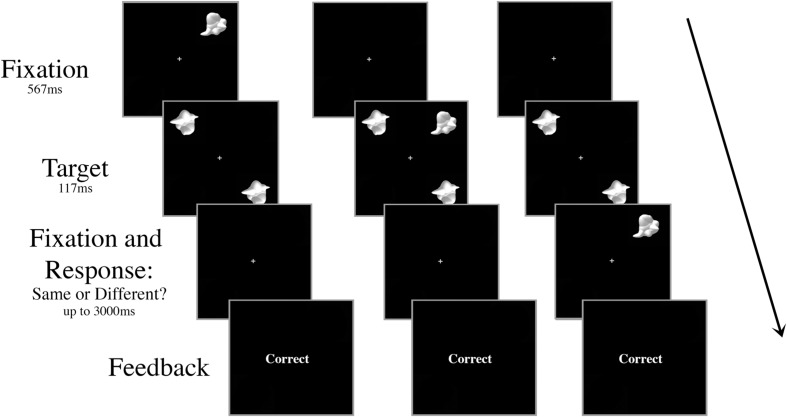
**Schematic of three peripheral distractor trial types in Experiment 2.** The targets and the distractor were always displayed for 117 ms regardless of SOA. The distractor appeared either **(A)** 267 ms or 117 ms prior to target onset, **(B)** simultaneously with target onset, or **(C)** 117 ms or 267 ms after target onset.

All aspects of the stimuli and apparatus were the same as Experiment 1, with the exception that there was no inconsistent distractor – all trials had the “consistent” smoothie distractor as the visual distractor – and the distractor appeared centrally on only half the trials. On the other half of the trials, the distractor appeared in the periphery at 6.5° eccentricity, but at a different location to the target stimuli (**Figure 4**). To ensure the participant knew which stimuli were the targets, the stimuli always appeared in the same configuration: targets in the upper left and lower right quadrants of the screen. When present, the peripheral distractor always appeared in the upper right quadrant of the screen.

The shortest distance between the two targets must be directly at fixation, and thus the peripheral distractor is presented a farther distance from the targets than the central distractor. However, the only way to completely control for target–distractor distance is to alter the location of the targets in peripheral trials, which introduces other possible confounds.

#### Procedure

Participants completed the same training as for Experiment 1.

The SOAs were identical to Experiment 1, but in Experiment 2 the distractor was always a smoothie and could be either central or peripheral. There were 80 trials for each of the ten conditions (central distractor at each SOA; peripheral distractor at each SOA) for a total of 800 trials in a session. As before, all of the trial types were intermingled randomly, but blocked so that the participant could rest every 100 trials.

## Results

Once again, our dependent variable was *d′* for target discrimination. A two-way repeated measures ANOVA on *d′* with the factors of distractor location (foveal, peripheral) and SOA (-267, -117, 0, +117, and +267 ms) revealed significant main effects of distractor location [*F*(1,18) = 7.978, *p* = 0.011] and SOA [*F*(4,72) = 6.56, *p* < 0.001], as well as a significant interaction [*F*(4,72) = 3.238, *p* = 0.017].

**FIGURE 5:**
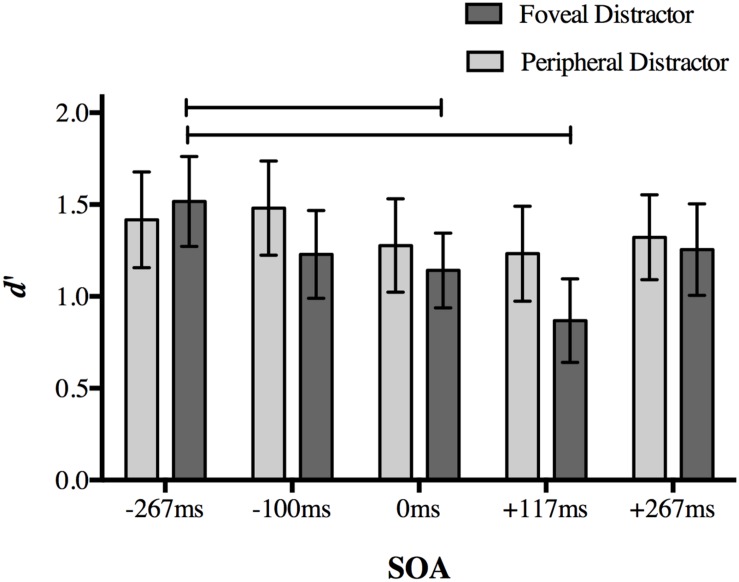
**The effect of distractor location on peripheral target discrimination in Experiment 2.** A foveal distractor appearing 117 ms after target onset or simultaneously with the targets disrupted discrimination accuracy more than a distractor presented at -267 ms SOA, replicating the main finding in Experiment 1. The timing of the distractor did not have an effect on discrimination accuracy when it was presented in the periphery. Lines indicate statistically significant differences between means after Bonferroni correction for 20 comparisons (*p* < 0.0025). Error bars represent 95% confidence intervals.

*Post hoc* analyses of the interaction using a Bonferroni correction for multiple comparisons (α = 0.05/20 = 0.0025) showed a replication of the effect found in Experiment 1. When a foveal distractor was presented 117 ms after target onset, behavioral performance was impaired compared to a foveal distractor presented at the baseline -267 ms SOA (*p* < 0.001; **Figure 5**). Differences in mean *d′* values compared to other SOAs did not survive correction (+117 ms SOA relative to 117 ms: *p* = 0.007; 0 ms: *p* = 0.012; +267 ms: *p* = 0.002).

Accuracy at 0 ms SOA in the foveal distractor condition was also less than baseline -267 ms SOA (*p* = 0.001). No other comparison approached significance after correction (*p* > 0.014).

When the distractor was presented in the periphery, differences in *d′* values between a distractor at -117 ms SOA and +117 ms SOA did not survive correction (*p* = 0.022), and no other comparisons approached significance (*p* > 0.05). Taken together, these results indicate that the effect found in Experiment 1 is limited to a distractor presented at the fovea.

## Discussion

In Experiment 1, we found that a visual distractor presented at fixation at +117 ms SOA disrupted behavioral performance more than distractors presented at other non-simultaneous SOAs. In our key condition, the stimuli of interest are no longer present and the distractor appears in an entirely different location from that of the target array. Despite this, we see significant impact of this distractor on the discrimination performance on peripheral objects. Critically, we observe a “recovery” in perceptual discrimination ability if the distractor is presented later in the course of a trial, suggesting that the behavioral disruption at +117 ms SOA is not simply a result of displaying a distractor object any time after stimulus onset, but reflects a critical timecourse for the disruption of the feedback phenomenon. This paradigm does not speak to the location in the brain involved in the interference with visual processing, but based on the collective work of Williams et al. (2008) and Chambers et al. (2013), we infer that it occurs at the foveal confluence.

In Experiment 2, we tested whether the disruptive effect depended on the retinotopic location of the visual distractor by adding a condition in which the visual distractor appeared in the periphery. We found that when the distractor was presented in the periphery, discrimination accuracy was similar across SOAs. In the same experiment, we were able to replicate our main finding from Experiment 1: a foveal distractor presented at +117 ms SOA disrupted behavioral performance more than a distractor presented at a very early SOA of -267 ms. A simultaneously presented foveal distractor also impaired discrimination accuracy relative to the baseline SOA.

What can account for the differential effect of distractor location on perceptual accuracy? In Experiment 2, the peripheral and foveal distractors were the same physical size, but cortical magnification results in different representational size in the cortex (Daniel and Whitteridge, 1961). The consequence of this is that the peripheral distractor would be represented in a smaller part of cortex than the central distractor in foveal cortex. This may result in a foveal distractor causing more interference overall than a peripheral distractor. In fact, distractors presented at fixation are more distracting than peripheral distractors, even when peripheral distractors are scaled for cortical magnification (Beck and Lavie, 2005), suggesting this is not the only factor at play.

Another possibility is that the central distractor drives stronger attentional capture than the peripheral distractor because it is presented at the midpoint between the targets. Williams et al. (2008) addressed this possibility with a control fMRI experiment. When the targets were placed in both upper left and right quadrants (or in both lower quadrants) of the display, they again found object category information at the foveal confluence, and no object information at the midpoint between targets. We used a very similar behavioral task, suggesting our effect is unlikely to be driven by the peripheral distractor simply falling outside the spotlight of attention.

For either explanation, if our result was driven by differences in cortical magnification or other non-specific alerting or attentional effects, we should expect an effect of distractor location for all SOAs, perhaps most evident when the distractor was presented simultaneously with the peripheral targets. Instead, the only SOAs where distractor location had a significant effect on behavior was at -117 ms pre-target onset and +117 ms post-target onset. Thus, the most parsimonious explanation is that the differential effect at +117 ms SOA reflects disruption of feedback processes, for example, through greater competition for attentional resources to ignore the central distractor.

The timing of our effect suggests that the foveal representation in visual cortex is recruited via feedback mechanisms for challenging peripheral object discrimination during a period ~200–340 ms post-target presentation. This timing is somewhat earlier than the critical timecourse described in Chambers et al. (2013). The timecourse described in these experiments are, however, consistent with other masking paradigms used to examine feedback effects on visual perception (Lamme et al., 2002; Wyatte et al., 2012), as well as other TMS studies targeting early visual areas at late latencies (e.g., +150 ms SOA in Koivisto et al., 2011), and TMS studies targeting higher visual areas at early latencies (e.g., -53 to -13 ms SOA; Vetter et al., 2013). The reasons for the discrepancy between the foveal distractor paradigm described here and (Chambers et al., 2013) are unclear, and may reflect differences in the behavioral tasks. For instance, the presentation duration of the target stimuli in Chambers et al. (2013) varied between participants so that the task was equally difficult for all participants. Here, the duration was maintained at 117 ms throughout the experiment, with some individual variance in overall accuracy. It is plausible that such differences could affect the exact timecourse of feedback: perceptually difficult tasks may take longer to be fed back to the foveal confluence. Alternatively, it may reflect a coarseness in our choices of SOAs; it will be important in future research to examine, for instance, overlapping SOAs from our +117 ms SOA condition out to +267 ms with shorter distractor presentation to allow a more fine-grained timecourse analysis.

What is the purpose of this feedback mechanism? One possibility is that foveal visual cortex may serve as a high-resolution buffer, or visual “scratchpad,” for storing task-relevant information for the purpose of increasing the precision of difficult perceptual decisions (Lee and Mumford, 2003). If this is the case, visual input at the fovea that contains information consistent with the perceptual task may not impair behavioral performance as much as an irrelevant distractor. Alternatively, it may be that a distractor that is more similar to the targets interferes with feedback processes, in which case an irrelevant distractor may be more easily ignored. Under either possibility, there should be a different effect of consistent versus inconsistent distractors on behavior. In Experiment 1, we manipulated the type of the distractor by using either a consistent (smoothie) or inconsistent (cubie) distractor relative to the target stimuli. We found no evidence that this influenced the magnitude of the effect. One possibility is that, despite coming from another category, the cubie distractor was still similar enough to the smoothie distractor that any congruency effects were masked (but see Yu and Shim, 2016). Alternatively, it may be that any information presented at the fovea provides feedforward input that then competes with the use of this region of cortex for other purposes.

Taken together, the results of these experiments suggest that a feedback mechanism critical for extra-foveal perception is specific to foveal space, corroborating results from previous studies using TMS (Chambers et al., 2013) and fMRI (Williams et al., 2008). These studies all indicate that foveal feedback aids the peripheral discrimination of shape in some way when shape is the task-relevant characteristic of the stimuli. Whether foveal feedback aids in the discrimination of other visual characteristics (e.g., color, orientation) remains to be explored. The behavioral paradigm described here provides a valuable method to investigate the way in which feedback influences visual perception in the periphery, paving the way for a deeper understanding of the purpose and functionality of the foveal feedback phenomenon.

## Author Contributions

All authors contributed to the study design. KW programmed the experiment and collected the data. KW analyzed the data under the supervision of AW, AR, and MW. KW drafted the manuscript, and MW, AR, and AW provided critical revisions. All authors approved the final version of the manuscript for submission.

## Conflict of Interest Statement

The authors declare that the research was conducted in the absence of any commercial or financial relationships that could be construed as a potential conflict of interest.
